# Determining the Effects of Cattle Grazing Treatments on Yosemite Toads (*Anaxyrus* [=*Bufo*] *canorus*) in Montane Meadows

**DOI:** 10.1371/journal.pone.0079263

**Published:** 2013-11-05

**Authors:** Susan K. McIlroy, Amy J. Lind, Barbara H. Allen-Diaz, Leslie M. Roche, William E. Frost, Rob L. Grasso, Kenneth W. Tate

**Affiliations:** 1 Environmental Science, Policy, and Management Department, University of California, Berkeley, Berkeley, California, United States of America; 2 USDA Forest Service, Pacific Southwest Research Station, Davis, California, United States of America; 3 Department of Plant Sciences, University of California Davis, Davis, California, United States of America; 4 Agriculture and Natural Resources Research and Extension Centers, Davis, California, United States of America; University of Sydney, Australia

## Abstract

Amphibians are experiencing a precipitous global decline, and population stability on public lands with multiple uses is a key concern for managers. In the Sierra Nevada Mountains (California, USA), managers have specifically identified livestock grazing as an activity that may negatively affect Yosemite toads due to the potential overlap of grazing with toad habitat. Grazing exclusion from Yosemite toad breeding and rearing areas and/or entire meadows have been proposed as possible management actions to alleviate the possible impact of cattle on this species. The primary objective of this study was to determine if different fencing treatments affect Yosemite toad populations. We specifically examined the effect of three fencing treatments on Yosemite toad breeding pool occupancy, tadpoles, and young of the year (YOY). Our hypothesis was that over the course of treatment implementation (2006 through 2010), Yosemite toad breeding pool occupancy and early life stage densities would increase within two fencing treatments relative to actively grazed meadows due to beneficial changes to habitat quality in the absence of grazing. Our results did not support our hypothesis, and showed no benefit to Yosemite toad presence or early life stages in fenced or partially fenced meadows compared to standard USDA Forest Service grazing levels. We found substantial Yosemite toad variation by both meadow and year. This variation was influenced by meadow wetness, with water table depth significant in both the tadpole and YOY models.

## Introduction

Scientists have documented a significant global amphibian decline [1,2], with over 40% of amphibian species currently considered threatened [3]. Potential drivers for this decline include habitat modifications and/or loss [4,5], disease [6], introduction of alien species [7], global warming [8], and pesticides [9]. In the Sierra Nevada Mountains of California (USA), cattle grazing has been identified by managers as a potential driver of amphibian decline [4,10]. State and Federal regulatory agencies consider nearly half of the 30 native amphibian species across the Sierra Nevada to be at risk of decline [4,10,11]. One species believed to have declined or disappeared from at least 50% of its historic range is the Yosemite toad (*Anaxyrus* [=*Bufo*] *canorus*) [12–14]. The Yosemite toad is a California Species of Special Concern, a USDA Forest Service Sensitive Species, and a candidate species for federal listing under the Endangered Species Act [4,10,11]. 

Yosemite toads use high montane and subalpine wet meadows between 1900 - 3500 m elevation for breeding and rearing [12,14,15]. They are active above ground for only about four months each year, with adults generally emerging from hibernation in May or June following snowmelt. Breeding then occurs for 2-4 weeks with oviposition sites at the shallow edges of pools or slow-moving streams [15,16]. These areas remain occupied by the egg and tadpole life stages for 6-8 weeks following breeding. After metamorphosis, the young of the year (YOY) generally remain close to the breeding pools until hibernation. Species success is heavily dependent on annual snowpack, as changing storm patterns and drought can delay or interrupt breeding and affect survival of juveniles and adults [12,17]. 

Cattle grazing on USDA Forest Service (hereafter Forest Service) lands is common across the Yosemite toad’s range, with nearly 40,000 head of cattle transported to Sierra Nevada national forests for summer grazing each year [18]. Managers have specifically identified grazing as an activity that may negatively affect Yosemite toads due to the potential overlap of grazing allotments with toad breeding pools and rearing areas in montane meadows [4,19]. Specific mechanisms that may degrade breeding pools and associated habitat include hoof trampling, which may fragment or widen pools and lead to water temperature increases, faster drying of pools and potential bird predation [20], consumption of herbaceous biomass, which may also increase temperatures and decrease escape cover [21,22], and deposition of nitrogenous waste, which may cause elevated ammonia, nitrite, and nitrate, all of which have been shown to decrease survival of amphibian embryos and larvae [23]. 

However, recent studies have shown that livestock grazing has either mixed effects [24] or no negative effects on amphibians [25,26]. For example, studies conducted in northeastern Oregon showed no significant effects of moderate grazing on Columbia spotted frog (*Rana luteiventris*) reproduction and short-term survival [25,27]. A companion study to our work examined Yosemite toad response to a gradient of livestock utilization in Sierra Nevada meadows and found no direct effect of grazing on Yosemite toad presence [26]. 

Due to the reported Yosemite toad population decline and its potential link to livestock grazing, the Forest Service developed alternative management strategies for confirmed areas occupied by Yosemite toads that were actively grazed. In 2001, a number of Sierra Nevada grazing permits were changed to non-use. Range managers are also considering fencing of occupied areas and/or a shortened grazing season such that cattle use of grazing allotments would be delayed until post-metamorphosis. Implementing any of these management strategies could affect other meadow-associated species (e.g., willow flycatchers, special status fish species) as well as cause significant economic hardship to the ranchers that depend upon high elevation meadows for summer forage. Due to a lack of quantitative data linking livestock grazing to Yosemite toad decline, the primary objective of this study was to determine if alternative fencing treatments would impact Yosemite toad populations. We specifically examined the effect of three fencing treatments on Yosemite toad breeding pool occupancy, tadpoles, and YOY. Our hypothesis was that over the course of treatment implementation (2006 through 2010), Yosemite toad breeding pool occupancy and early life stage densities would increase within two fencing treatments relative to actively grazed meadows due to beneficial changes to habitat quality in the absence of grazing. 

## Methods

### Ethics statement

This observational field study was conducted in collaboration with the USDA Forest Service, so all permissions for site access were granted and no permits were required. 

### Study area and design

**Figure 1:**
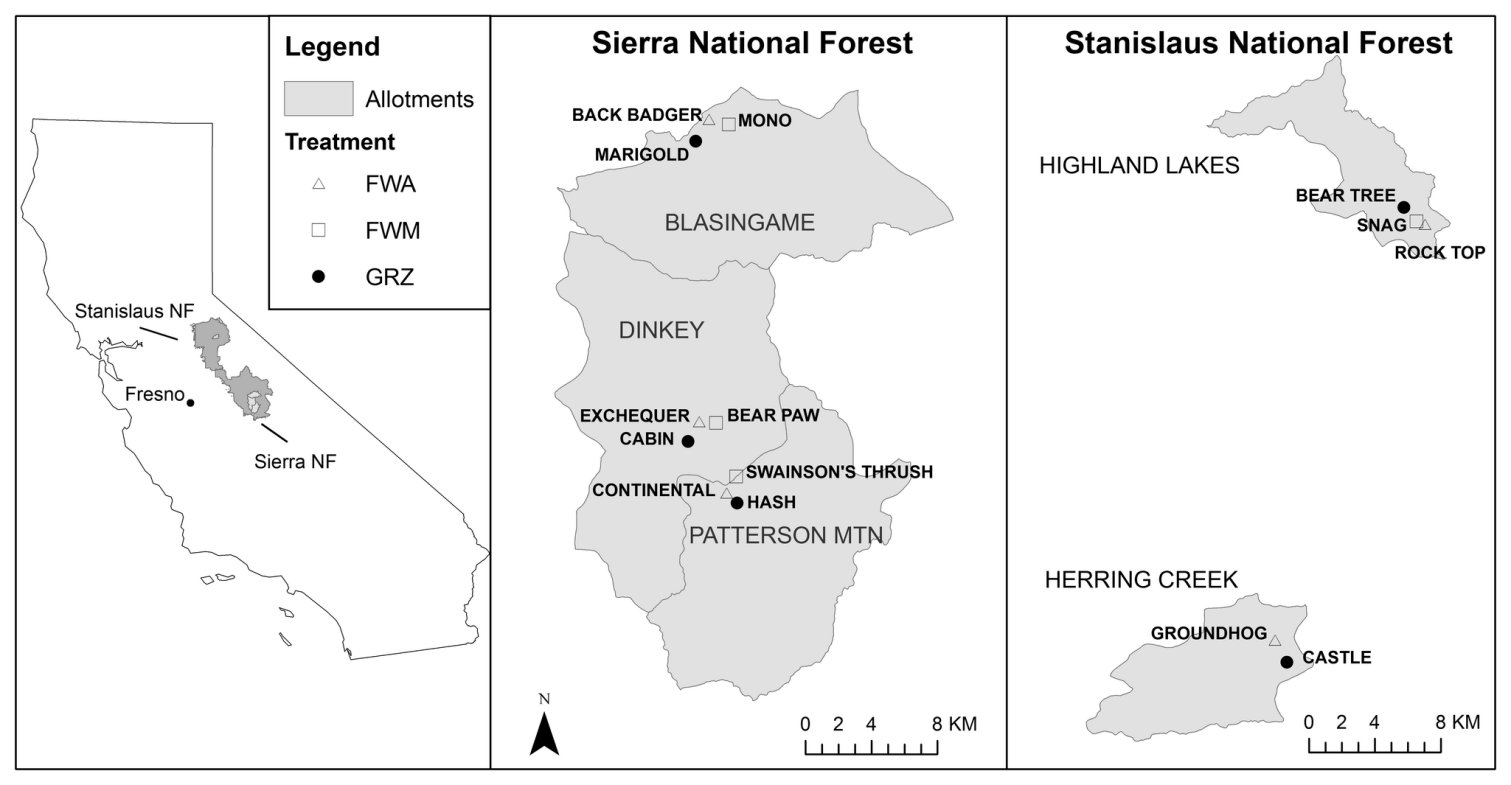
The study area, showing the Stanislaus and Sierra National Forests, grazing allotments, and meadows by treatment.

The study area included 14 meadows on the western slope of the central Sierra Nevada Mountains of California (USA), with five meadows on the Stanislaus National Forest (38° N, 120° W), and nine on the Sierra National Forest (37° N, 119° W; Figure 1). Meadows ranged in size from 0.7-23.3 ha and in elevation from 2,113-2,717 m (Table 1). Long-term annual precipitation averages 121 cm across the meadows [28]. The majority of precipitation falls as snow from October to April and snowmelt generally occurs in May or June. When Yosemite toads emerge from hibernation to breed and lay eggs, meadows are characterized by shallow flooded areas. Flooding recedes as the summer progresses, resulting in pools ranging in size from > 100 m^2^ in the spring to more discrete pools (~1 m^2^) later in the season [26]. All study meadows were identified as wet (0-30 cm depth to water table) or mesic (30-80 cm water table depth) based on both literature review [29,30] and field visits [31]. Meadow soils were classified as Mollisols and Inceptisols with Histosols found in the wettest meadow areas [26]. Meadow vegetation is characterized predominantly by perennial herbaceous species such as *Carex nebrascensis*, *Eleocharis pauciflora*, *Juncus oxymeris*, and *Polygonum bistortoides*. Dominant forest vegetation surrounding the meadows includes *Abies concolor, Abies magnifica*, *Pinus contorta*, and *Pinus jeffreyi.*

**Table 1:** Study meadow environmental characteristics.

**Treatment Meadows**	**Forest**	**Grazing Allotment**	**Elevation (m)**	**Survey Area (ha)**	**Depth to Water Table (cm) 2006-2010**	**Water Year Precip. (cm) 2006-2010**	**Utilization (%) 2006-2008**	**Herbaceous Biomass (kg/ha 2006-2008**
*Fence Whole Meadow (FWM)*								
Bear Paw	Sierra NF	Dinkey	2271	1.5	6.8 (1.3)	108 (40)	17 (4)	2782 (166)
Mono	Sierra NF	Blasingame	2659	2.9	4.7 (1.1)	112 (45)	7 (2)	1582 (139)
Snag	Stanislaus NF	Highland Lakes	2592	0.8	37.6 (2.8)	131 (38)	10 (3)	2983 (387)
Swainson's Thrush	Sierra NF	Patterson Mtn.	2258	0.9	11.5 (1.3)	100 (37)	10 (3)	2911 (157)
*Fence Breeding Area (FBA)*								
Back Badger	Sierra NF	Blasingame	2519	1.2 [0.5]	31.4 (3.6)	105 (43)	17 (4)	1687 (112)
Continental	Sierra NF	Patterson Mtn.	2155	4.2 [1.1]	11.6 (1.2)	100 (37)	5 (1)	3461 (281)
Exchequer	Sierra NF	Dinkey	2226	6.4 [3.2]	17.2 (1.9)	99 (38)	15 (3)	3275 (241)
Groundhog	Stanislaus NF	Herring Creek	2589	3.4 [0.3]	41.5 (2.9)	136 (43)	7 (2)	4116 (263)
Rock Top	Stanislaus NF	Highland Lakes	2613	0.9 [0.2]	46.5 (3.9)	131 (38)	7 (3)	4073 (392)
*Grazed (GRZ)*								
Bear Tree	Stanislaus NF	Highland Lakes	2542	2.3	46.4 (3.6)	131 (38)	43 (5)	2969 (258)
Cabin	Sierra NF	Dinkey	2134	2.6	35.7 (2.3)	92 (35)	41 (4)	3536 (234)
Castle	Stanislaus NF	Herring Creek	2679	5.9	33.5 (3.5)	137 (43)	31 (6)	3507 (295)
Hash	Sierra NF	Patterson Mtn.	2122	2.3	16.8 (1.5)	100 (37)	30 (5)	3521 (302)
Marigold	Sierra NF	Blasingame	2543	8.5	20.0 (1.7)	105 (43)	13 (4)	2418 (242)

Bracketed values in survey area column indicate total fenced area for FBA treatment meadows. Values for water table depth, water year precipitation, utilization, and herbaceous biomass are mean annual estimates with 1 standard error of mean in parenthesis.

Meadows were located within five active grazing allotments that were 5,700 -27,000 ha in size with cattle numbers per allotment ranging from 156-235 cow-calf pairs. Both National Forests have had long-term livestock use and current grazing under Forest Service Standards and Guidelines requires grazing utilization levels to not exceed 40% [19]. Cattle grazing began in late June/early July and continued into September in all study years. 

In 2005 (pilot year) potential meadows were randomly selected and field surveys were conducted to confirm both actively breeding Yosemite toad populations as well as current grazing that was typical of Forest Service grazing standards. We used a randomized complete block design to assign each of the selected meadows to one of three fencing treatments (Table 1). Allotments were treated as blocks and each allotment had the following treatments applied: 1) compliance with annual grazing standards and guidelines (GRZ) of no more than 40% use [19]; 2) fencing of Yosemite toad breeding areas (FBA); and 3) fencing the whole meadow to exclude cattle (FWM; Figure 1). The FWM treatment was designed to eliminate any direct physical and/or depositional (e.g. increased nutrient loading) impacts on Yosemite toad habitat and populations while the FBA treatment provided a less restrictive option. This treatment would allow for some grazing to continue on a meadow but would presumably protect Yosemite toad habitat from any direct grazing impacts. A meadow on the Stanislaus National Forest was removed from the study due to improper treatment implementation, so one allotment (Herring Creek) lacks the FWM treatment. 

### Field methods

Within each meadow we established 2-4 transects that were aligned perpendicular to water flow and/or gradient. Along transects we placed 10 groundwater wells to measure water table depth in areas that represented dominant meadow vegetation [31]. Within 3 m of each well we placed a livestock utilization cage and paired plot. Cattle utilization (%) and herbaceous biomass production (kg/ha) were monitored using the comparative yield method at the 10 paired plots within each meadow [32,33]. Water table wells were measured at least monthly except in 2010 when fewer measurements were taken due to broken wells. Herbaceous biomass production (kg/ha) and utilization (%) were measured following livestock removal in September (2006-2008). 

We used continuous parallel transects [34] to survey each study meadow for Yosemite toad breeding pools each year. This method provided nearly complete coverage of each meadow, but it excluded more upland and dry areas. These areas were not surveyed and they were not used for calculation of densities of any lifestage. A breeding pool was defined as occupied if it had Yosemite toad eggs and/or tadpoles. Each breeding pool was staked and given a unique number in 2005. Additional pools without Yosemite toad eggs or tadpoles present but with similar characteristics to occupied pools were characterized as unoccupied. All pools were checked for presence during all subsequent years and any newly occupied pools were staked and given a unique number. Occupied pools and a paired subset of unoccupied pools (up to three per meadow) were measured intensively throughout the study, including collection of detailed habitat data. 

Tadpole counts were done using stratified random ‘hoop counts’ conducted during one visit per summer for each of the occupied breeding pools. Hoops were ¼ m^2^ circles of plastic tubing that were randomly placed within two strata (sparse and dense areas of tadpoles) defined in each pool. The strata were visually determined by consensus among field crew members and roughly corresponded to >5 (dense) and <5 (sparse) tadpoles per m^2^. The relative survey effort per strata was determined based on pilot study data, with a higher percentage of hoops placed in dense areas than sparse areas to improve precision of estimates. The total number of hoop counts varied depending on the pool area but a minimum of two hoops were placed in each strata within each pool. Over the course of the study, the per pool maximum hoop counts were 23 dense hoops and 75 sparse hoops [35]. In the pilot study year, each hoop was counted by two different observers to confirm the precision of density estimates. Individual observer counts had low variance, so for the remainder of the study only one observer counted each hoop. Habitat conditions (water depth and temperature, detritus depth, dominant cover and substrate, and flow) were recorded for each occupied and paired unoccupied pool. For calculations of total tadpole counts, total hoop counts for each breeding pool were first calculated incorporating the two strata and then pool counts were summed to derive a count for the entire meadow. YOY surveys were conducted using continuous parallel transects at each study meadow. Each YOY was batch marked with a meadow color and year color using visible implant elastomer to avoid double counting. Annual counts of YOY for each meadow were calculated as the average of two counts spaced approximately three weeks to one month apart within each year.

### Data analysis

We used generalized linear mixed model regression analysis [36] to test for meadow grazing treatment effects on: 1) annual proportion of occupied breeding pools per meadow, 2) annual tadpole density, and 3) annual YOY density for the 2006 through 2010 study years. The number of occupied breeding pools was analyzed as a binomial (number of occupied pools / total number of surveyed pools) with a logit link function. The total number of surveyed pools was calculated as the total number of pools in which breeding ever occurred within a meadow, across all years, plus the paired unoccupied pools surveyed intensively throughout the study. Tadpole and YOY density were analyzed as count response variables using the log link function (Poisson family) with robust standard errors for overdispersion [37].

In accordance with the experimental design, the specific test of the hypothesis is based upon the significance of the year by treatment interaction (year × trt) and the relative pattern of response among treatment meadows over time. The remaining fixed effect variables were covariates to account for inherent differences in toad densities or number of occupied pools among treatment groups at the outset of the study (trt), year to year variation (year), and meadow wetness (mean depth to water table; averaged for each meadow across all years [2006-2010]). The area of each meadow surveyed for Yosemite toads was used as an offset variable to account for unequal meadow size, essentially resulting in an analysis of densities. To account for repeated measures on each meadow and blocking by allotment, meadow ID and allotment ID were specified as random effects. When significant fixed effects were found in models, we conducted pairwise comparisons to clarify the source of the differences and we included a Tukey adjustment for these multiple t-tests. 

## Results

### Environmental conditions

During the study, annual precipitation ranged from 53% to 138% of the 121 cm long-term average (2007 and 2006, respectively). Annual precipitation during the other three study years was 76% (2008), 91% (2009), and 110% (2010) of the long-term average. Water table values varied with annual precipitation but were overall stable in these hydric and mesic meadows. Mean water table depth across the 14 meadows averaged 25.6 cm (± 0.9 SE) and ranged from an average low depth of 31.1 cm (± 1.8 SE) in 2007 to a high of 22.3 cm (± 1.9 SE) in 2006 (Table 1). Among treatments, mean water table depth was very similar between the GRZ (30.2 cm ± 1.4 SE) and FBA (29.5 cm ± 1.5 SE) meadows but the FWM sites were significantly wetter (15.2 cm ± 1.3 SE). Average annual utilization by meadow ranged from 5% (± 1% SE) at Continental Meadow to 43% (± 5%) at Bear Tree Meadow, with a mean across meadows of 17% (± 1 % SE) (Table 1). Among treatments, both the FBA and FWM meadows had an average utilization of 11 % (± 1% and 2% SE, respectively) while the GRZ meadows had an overall use of 31% (± 2% SE). The recorded utilization within fenced areas for the FBA and FWM meadows is likely due to small spatial scale variation between the caged and uncaged paired plots, which in some cases showed a minimal amount of utilization even in the clear absence of herbivorous grazing. Additionally, native deer grazing was commonly observed within fences and there were also a few confirmed cattle trespass events [38], both of which likely contributed to detectable utilization values within fences. Herbaceous biomass production averaged 3,041 kg/ha (± 78 SE) across meadows with a range of 1,582 (± 139) kg/ha at Mono Meadow to 4,116 (± 263 SE) kg/ha at Groundhog Meadow (Table 1). Across treatments biomass was lowest across the FWM meadows (2,542 kg/ha ± 148 SE) but comparable across FBA (3,317 kg/ha ± 147 SE) and GRZ (3,183 kg/ha ± 138 SE) meadows. 

### Yosemite toads

**Figure 2:**
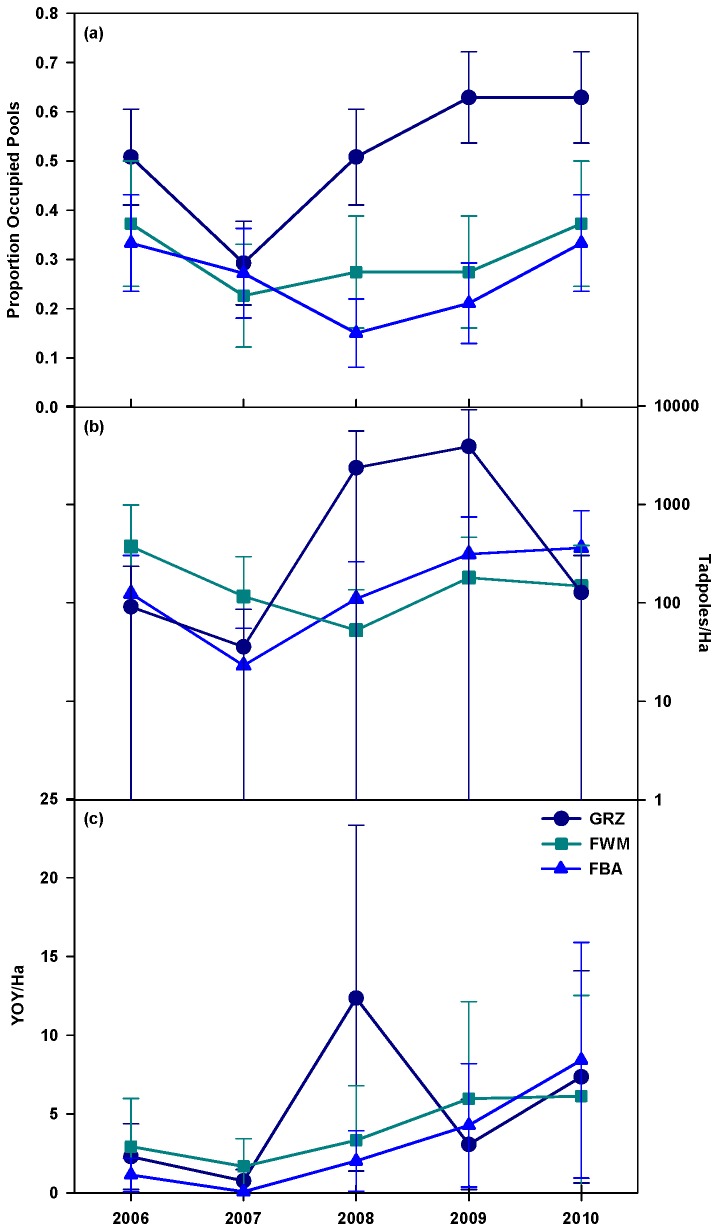
Yosemite toad response to grazing treatments over time (year x trt): (a) pool occupancy rates, (b) tadpoles (log scale), and (c) YOY. The mean values (± 1 SE) in these figures are least square means which have been adjusted for meadow wetness and meadow survey area.

Yosemite toad presence and density were highly variable among years and grazing treatments over the course of the study (Figure 2). Unadjusted (for meadow wetness/depth to water table) breeding pool occupancy rates across all years and treatments ranged from 0-100% with a mean of 36% (± 3% SE). The FBA and FWM treatments had a similar percentage of occupied pools, with 26% (± 4% SE) and 32% (± 4% SE), respectively. The GRZ meadows had the highest percentage of occupied pools with 49% (± 4% SE). The individual meadow with the highest occupied pools averaged across years was Castle (65% ± 9% SE), although Marigold had similar results (62% ± 9% SE). Unadjusted mean tadpole density across the study meadows was 1,813 per ha (± 358 SE) and ranged from 0-16,370 tadpoles/ha. Among treatments mean tadpole density ranged from 850 (± 251 SE) in the FBA meadows to 3,110 (± 1,134 SE) in the FWM treatment, while the GRZ meadows averaged 1,747 ± (565 SE). Unadjusted YOY densities were much lower, with a mean of 19 per ha (± 2 SE) and a range from 0-145 YOY/ha. Again, the FBA treatment had the lowest density with 9 (± 3 SE) YOY/ha while the FWM treatment had 30 (± 7 SE) YOY/ha and the GRZ meadows had 19 (± 8 SE) YOY/ha. Mono meadow had the highest tadpole (8,037 ± 2,221 SE) and YOY densities (59 ± 18 SE) compared to the other study meadows. It is likely that the high water table depth (4.7 cm ± 1.1 SE) at Mono played a key role in supporting Yosemite toad early life stages. 

The fixed effect (year x trt) that directly addressed our hypothesis and would have indicated differences among the treatments over time was not significant for any of the three response variables (Table 2). In the pool occupancy model, treatment was a significant fixed effect due to several pairwise differences (Figure 2). Specifically, there was a higher proportion of occupied pools for the GRZ treatment compared to the FBA treatment in 2008 and 2009; all other pairwise tests were not significant. Water table was significant in both the tadpole and YOY models. The relationship was negative in both models, such that there were lower densities at meadows with greater depths to water table (dryer meadows). Year was also significant in the YOY model due to several pairwise differences among years (Figure 2). Across the GRZ meadows, there were significantly higher densities of YOY in 2008 compared to 2007. Within the FBA meadows, YOY densities were significantly higher in both 2009 and 2010 compared to 2007. Water year 2007 was the driest of the study period and we suspect these differences in YOY density may be due in part to the more extended snowmelt period of 2009 and 2010 with meadows holding moisture later in the summer compared to 2007. 

**Table 2:** Summary of generalized linear mixed modeling.

**Treatments by Life Stage**	**Modeling Results F-value (p-value)**
*Pool occupancy*	
Year	2.00 (0.110)
Trt	**5.58 (0.007)**
Year x Trt	1.03 (0.429)
Water Table Depth	0.96 (0.333)
*Tadpoles*	
Year	2.62 (0.053)
Trt	0.43 (0.663)
Year x Trt	1.61 (0.161)
Water Table Depth	**5.81 (0.037)**
*Young of the Year*	
Year	**5.24 (0.003)**
Trt	0.81 (0.469)
Year x Trt	1.21 (0.327)
Water Table Depth	**7.49 (0.021)**

Significant results (p = 0.05) are in bold.

## Discussion

Over the course of the five year study, we did not detect a significant year by treatment effect of fencing on any Yosemite toad response variable. As implemented in this study, we found no benefit of fencing to Yosemite toad populations. Instead, substantial spatial and temporal population variation was influenced by meadow wetness, with water table depth significant in both the tadpole and YOY models. Previous research has shown that more than 50% of Yosemite toad subadults and adults are found in wet meadow bottoms [39], and our results support these findings. The hydric and mesic meadows in this study undoubtedly provide more suitable habitat for amphibians because they remain wet for an extended period of time, thereby reducing the risk of pools drying prior to the completion of metamorphosis. 

Our results do not support previous studies that found a negative impact of grazing on amphibian populations [21,22,24,40]. However, many of these studies were conducted in more intensively grazed systems with a comparison between high levels of grazing and the absence of grazing. For example, Burton et al. [24] found that relative abundance of green frog (*Rana clamitans*) metamorphs was lower at wetlands across the Cumberland Plateau (Tennessee, USA) that had cattle access compared to those where cattle were not present, but the study wetlands were intensively grazed year-long with an average density of 132 cattle/ha per month. For comparison, the five allotments in this study range in size from 5,700-27,000 ha and each have 156-235 cow-calf pairs distributed across them during about a four month grazing season. Although cattle have been shown to preferentially select meadows and riparian areas due to favorable biotic (e.g. forage quality and quantity, plant composition, and plant morphology) and abiotic conditions (e.g. slope, distance to water, topography [41,42];, their densities in the meadow systems in this study did not approach those in more intensively grazed systems. 

One management strategy that should not be considered for Yosemite toad conservation is partial meadow fencing as it was implemented in this study. Allowable livestock use as outlined in the Sierra Nevada Forest Plan Amendment [19] is 40%. We consistently found utilization above allowable use outside fenced areas at four of the five FBA treatment meadows. Because this treatment reduced the area of meadow available for grazing, it resulted in higher cattle density and increased utilization outside the fences [38]. Therefore, not only did partial fencing provide no benefit to Yosemite toads, this treatment resulted in higher utilization than current standards. This could result in negative grazing effects such as changes in vegetation species composition and abundance, increases in mechanical damage, and potential harm to other organisms that depend upon meadows for habitat. 

The known range of Yosemite toads spans five national forests and two national parks, each with federal and individual mandates to support multiple uses. Different land uses may impact Yosemite toads during multiple life stages. For instance, activities such as timber harvest may actually increase potential habitat by precluding conifer encroachment into breeding meadows and increasing water yield [43]. Cameras used in a companion study documented off-road-vehicle use directly through breeding habitat in Back Badger Meadow [38], which could negatively impact populations. Furthermore, Yosemite toads live only part of their life cycle in meadows, and they have been shown to move long distances into upland areas not necessarily associated with water sources [39,44]. Due to the complexity of Yosemite toad habitat needs and the variety of land uses that may impact them, continued monitoring and modeling of all toad life stages is critical for species conservation and success. 

## References

[1] Houlahan JE, Findlay CS, Schmidt BR, Meyer AH, Kuzmin SL (2000) Quantitative evidence for global amphibian population declines. Nature 404: 752–755. doi:10.1038/35008052. PubMed: 10783886. 10783886

[2] Stuart SN, Chanson JS, Cox NA, Young BE, Rodrigues ASL et al. (2004) Status and trends of amphibian declines and extinctions worldwide. Science 306: 1783–1786. doi:10.1126/science.1103538. PubMed: 15486254. 15486254

[3] IUCN Red List of Threatened Species. Version 2013.1. Available: www.iucnredlist.org. Accessed 2013 Oct 18

[4] USDI (2002) USDI Fish and Wildlife Service. Endangered and threatened wildlife and plants; 12 month finding for petition to list Yosemite toad. Fed Regist 67(237): 75834-75843.

[5] Cushman SA (2005) Effects of habitat loss and fragmentation on amphibians: A review and prospectus. Biol Conserv 128: 231–240.

[6] Daszak P, Cunningham AA, Hyatt AD (2003) Infectious disease and amphibian population decline. Divers Distrib 9: 141–150. doi:10.1046/j.1472-4642.2003.00016.x.

[7] Knapp RA, Matthews KR, Sarnelle O (2001) Resistance and resilience of alpine lake fauna to fish introductions. Ecol Monogr 71: 401–421. doi:10.1890/0012-9615(2001)071[0401:RAROAL]2.0.CO;2.

[8] Pounds JA, Bustamente MR, Coloma LA, Consuegra JA, Fogden MPL et al. (2006) Widespread amphibian extinctions from epidemic disease driven by global warming. Nature 439: 161–167. 16407945 10.1038/nature04246

[9] Sparling DW, Cowman D (2003) Amphibians and pesticides in pristine areas. 257–264. In: Linder G, Krest SK, Sparling DM, eds. Amphibian Decline: An Integrated Analysis of Multiple Stressor Effects. Society of Environmental Toxicology and Chemistry (SETAC), Pensacola, FL.

[10] USDI (2004) Endangered and threatened wildlife and plants; review of species that are candidates or proposed for listing as endangered or threatened; annual notice of findings on resubmitted petitions; annual description of progress on listing actions. Fed Regist 69(86): 25876–24904.

[11] California Department of Fish and Game (2011) California Natural Diversity Database Special Animals. Available: http://www.dfg.ca.gov/ biogeodata/ cnddb/ pdfs/ spanimals.pdf (January). Accessed on 10/13/2012

[12] Jennings MR, Hayes MP (1994) Amphibian and reptile species of special concern in California: final report to California Department of Fish and Game, Inland Fisheries Division. Rancho Cordova: California Department of Fish and Game. 255 pp.

[13] Drost CA, Fellers GM (1996) Collapse of a regional frog fauna in the Yosemite area of the California Sierra Nevada, USA. Conserv Biol 10: 414–425. doi:10.1046/j.1523-1739.1996.10020414.x.

[14] Jennings MR (1996) Status of amphibians. In: Sierra Nevada Ecosystem Project: final report to Congress. Davis. University of California, Centers for Water and Wildland Resources pp 921–944.

[15] Karlstrom EL (1962) The toad genus *Bufo* in the Sierra Nevada of California. University of California Publications in Zoology 62:1–104.

[16] Kagarise Sherman C (1980) A comparison of the natural history and mating system of two anurans: Yosemite toads (*Bufo**canorus*) and Black toads (*Bufo**exsul*). PhD Dissertation, University of Michigan, Ann Arbor, Michigan.

[17] Kagarise Sherman C, Morton ML (1993) Population declines of Yosemite toads in the eastern Sierra–Nevada of California. J Herpetol 27: 186–198. doi:10.2307/1564935.

[18] Derlet RW, Richards JR, Tanaka LL, Hayden C, Ka Ger et al. (2012) Impact of summer cattle grazing on the Sierra Nevada Watershed: aquatic algae and bacteria. J Environ Public Health. Vol 2012, Article ID: 760108, 7 p PubMed : 22505950 10.1155/2012/760108 PMC3312331 22505950

[19] USDA (2001) US Department of Agriculture. Sierra Nevada Forest Plan Amendment: final environmental impact statement and record of decision. Vallejo, CA, USA: Department of Agriculture Forest Service, Pacific Southwest region.

[20] Sherman CK, Morton ML (1984) The toad that stays on its toes. Natural History 93: 72-78.

[21] Healey M, Thompson D, Robertson A (1997) Amphibian communities associated with billabong habitats on the Murrumbidgee floodplain, Australia. Aust J Ecol 22: 270–278. doi:10.1111/j.1442-9993.1997.tb00672.x.

[22] Jansen A, Healey M (2003) Frog communities and wetland condition: relationships with grazing by domestic livestock along an Australian floodplain river. Biol Conserv 109: 207–219.

[23] Jofre MB, Karasov WH (1999) Direct effect of ammonia on three species of North American anuran amphibians. Environ Toxicol Chem 18: 1806–1812. doi:10.1002/etc.5620180829.

[24] Burton EC, Gray MJ, Schmutzer AC, Miller DL (2009) Differential responses of postmetamorphic amphibians to cattle grazing in wetlands. J Wildl Manag 73: 269–277. doi:10.2193/2007-562.

[25] Bull EL, Hayes MP (2000) Livestock effects on reproduction of the Columbia spotted frog. J Range Manag 53: 291–294. doi:10.2307/4003434.

[26] Roche LM, Allen-Diaz BH, Eastburn DJ, Tate KW (2012) b. Cattle grazing and Yosemite toad (*Bufo* * canorus* Camp) breeding habitat in Sierra Nevada meadows. Rangeland Ecol Manag 65: 56–65.

[27] Adams MJ, Pearl CA, McCreary B, Galvan SK, Wessell SJ et al. (2009) Short-term effect of cattle exclosures on Columbia spotted frog (*Rana* *luteiventris*) populations and habitat in northeastern Oregon. J Herpetol 43: 132–138. doi:10.1670/08-016R2.1.

[28] Daly C, Gibson WP, Taylor GH, Johnson GL, Pasteris P (2002) A knowledge-based approach to the statistical mapping of climate. Clim Res 22: 99–113. doi:10.3354/cr022099.

[29] Chambers JC, Blank RR, Zamudio DC, Tausch RJ (1999) Central Nevada riaprain areas: physical and chemical properties of meadow soils. J Range Manag 52: 92-99. doi:10.2307/4003497.

[30] Weixelman DA, Zamudio DC, Zamudio KA (1996) Central Nevada riparian field guide. USDA Forest Service, Intermountain Region, Ogden. R4-ECOL-96-01.

[31] McIlroy SK, Allen-Diaz BH (2012) Plant community distribution along water table and grazing gradients in montane meadows of the Sierra Nevada Range (California, USA).Wetlands Ecol Manag 20: 287–296. doi:10.1007/s11273-012-9253-7.

[32] Interagency Technical Team (1996) Utilization studies and residual measurements. Denver, CO, USA: US Department of the Interior, Bureau of Land Management, National Applied Resources Science Center, Interagency Technical Reference, Report BLM/RS/ST-96/004. 164 p

[33] McIlroy SK (2008) Identifying ecological patterns and processes in montane meadows of the Sierra Nevada range. PhD dissertation Berkeley, CA, USA University of California

[34] Sadinsky W (2004) Final Report to the Yosemite Fund on Amphibian Declines and Causes. USDI. Yosemite: National Park Service National Park

[35] Lind AJ, Grasso R, Nelson J, Vincent K, Liang C (2011) Determining the effects of livestock grazing on Yosemite toads (*Bufo* *canorus*) and their habitat: final report addendum to USDA Forest Service Region 5. Vallejo, CA, USA: USDA Forest Service.Region 5: 25.

[36] Institute SAS. Inc. (2008) SAS/STAT® 9.2 User’s Guide. Cary, NC: SAS Institute Inc

[37] Rabe-Hesketh S, Skrondal A (2008) Multilevel and longitudinal modeling using stata. College Station: Stata Press. 562 p.

[38] McIlroy SK, Allen-Diaz BH, Berg AC (2011) Using digital photography to examine grazing in montane meadows. Rangeland Ecol Manag 64: 187–195. doi:10.2111/REM-D-09-00130.1.

[39] Morton ML, Pereyra ME (2010) Habitat use by Yosemite toads: life history traits and implications for conservation. Herpetological. Conserv Biol 5: 388–394.

[40] Schmutzer AC, Gray MJ, Burton EC, Miller DL (2008) Impacts of cattle on amphibian larvae and the aquatic environment. Freshw Biol 53: 2613–2625. doi:10.1111/j.1365-2427.2008.02072.x.

[41] Bailey DW, Gross JE, Laca EA, Rittenhouse LR, Coughenour MB et al. (1996) Mechanisms that result in large herbivore grazing distribution patterns. J Range Manag 49: 386–400. doi:10.2307/4002919.

[42] George M, Ganskopp MDC, Bailey D, Borman M, Surber G et al. (2007) Factors and practices that influence cattle distribution. Davis, CA, USA: University of California Division of Agriculture and Natural Resources. Rangeland Management Series, Publication 8217, p 20.

[43] Semlitsch RD, Todd BD, Blomquist SM, Calhoun AJK, Whitfield Gibbons J et al. (2009). . . Effects of timber harvest on amphibian populations: understanding mechanisms for forest experiments 59 BioScience pp. 853–862. PubMed: 20201181.

[44] Liang C (2010) Habitat modeling and movements of the Yosemite toad (*Anaxyrus* (=*Bufo*) *canorus*) in the Sierra Nevada, California. Ph.D. Dissertation, University of California.

